# Hand, Foot, and Mouth Disease in a Patient With Psoriasis: A Case Report

**DOI:** 10.7759/cureus.80348

**Published:** 2025-03-10

**Authors:** João A Martins, Catarina Morais, Pedro Ferreira, Andreia Baptista

**Affiliations:** 1 Family Medicine, Unidade Local de Saúde-Almada Seixal, Almada, PRT

**Keywords:** case reports, enterovirus, hand foot and mouth disease, primary healthcare, psoriasis

## Abstract

Hand, foot, and mouth disease (HFMD) is a viral infection commonly found in children but rare in adults. Patients with chronic skin conditions may worsen after an inoculation with an infectious agent with cutaneous tropism. The present case reports a 48-year-old male patient with a history of plaque psoriasis, with no identifiable epidemiological contacts, who presented at a primary health care consultation with painful vesicles, blisters, and pustules in the perioral region, elbows, hands, and soles of the feet. He was also observed in the hospital emergency department, where blood tests confirmed an enterovirus A71 infection. The patient was subsequently followed up in a dermatology consultation. This case report emphasizes the need for health professionals to consider HFMD in adults with unexplained cutaneous lesions with oral symptoms, especially when chronic dermatological diseases are present, due to the risk of their worsening.

## Introduction

Hand, foot, and mouth disease (HFMD) is a highly contagious viral infection, primarily affecting children under the age of 10 [1,2]. This condition is mostly caused by coxsackieviruses, though other enteroviruses can also be responsible [2]. Typical symptoms include fever, malaise, odynophagia, anorexia, and the development of painful skin lesions on the hands, feet, oral cavity, and other parts of the body [3]. Transmission occurs through direct contact with bodily secretions such as saliva, feces, or nasal secretions [2]. Although generally mild and self-limiting in children, there is a rising incidence of HFMD in adults, presenting particular diagnostic and management challenges [4,5]. Less than 1% of infections in adults are symptomatic, yet they exhibit a wide variability in symptoms, ranging from constitutional symptoms and typical skin lesions to severe presentations involving the central nervous system or the heart [6]. In patients with chronic dermatological diseases, Koebner phenomenon may occur with the worsening of their underlying condition and the need for therapeutic adjustment [7]. For these reasons, it is important for healthcare professionals to consider HFMD in the differential diagnosis of adults who present with febrile or post-febrile skin lesions, in order to avoid potential diagnostic errors, especially if there is a risk of exacerbation of a known chronic skin disease. Here, we describe a 48-year-old male patient with plaque psoriasis, previously controlled in a primary health care setting, with disease exacerbation due to HFMD.

## Case presentation

**Figure 1:**
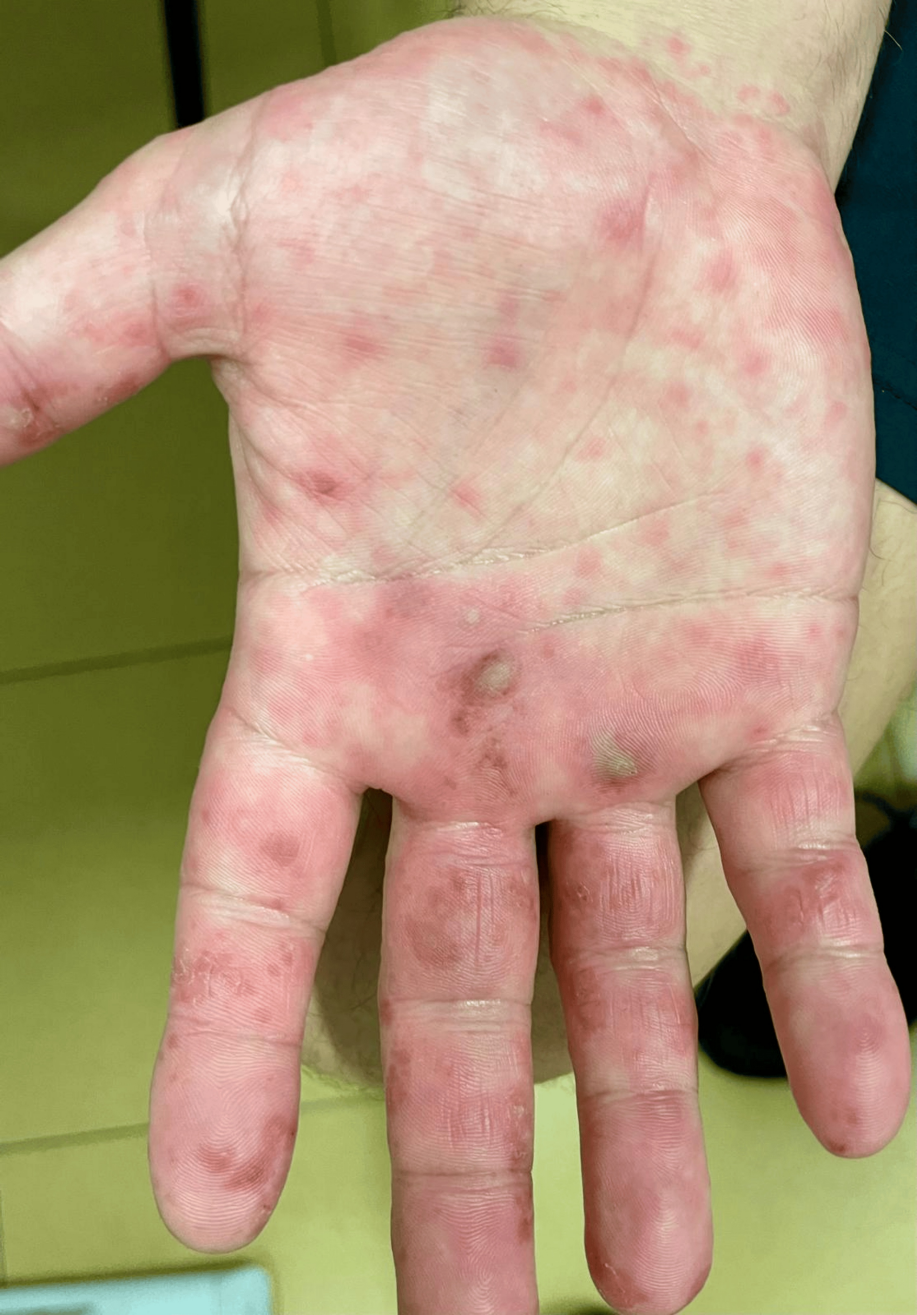
Vesicles, blisters and pustules on the palmar side of the right hand.

**Figure 2:**
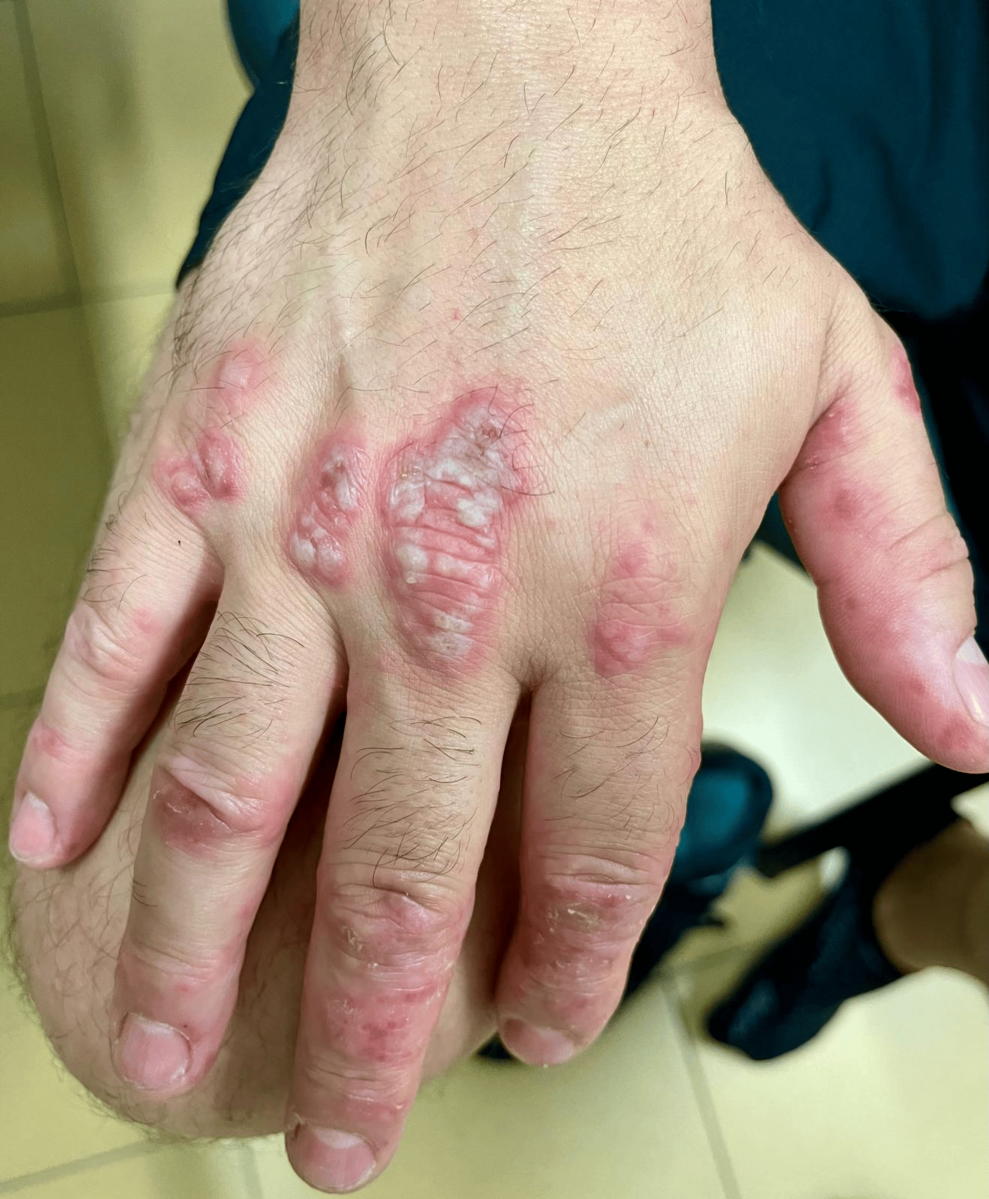
Vesicles, blisters and pustules on the dorsal side of the right hand.

**Figure 3:**
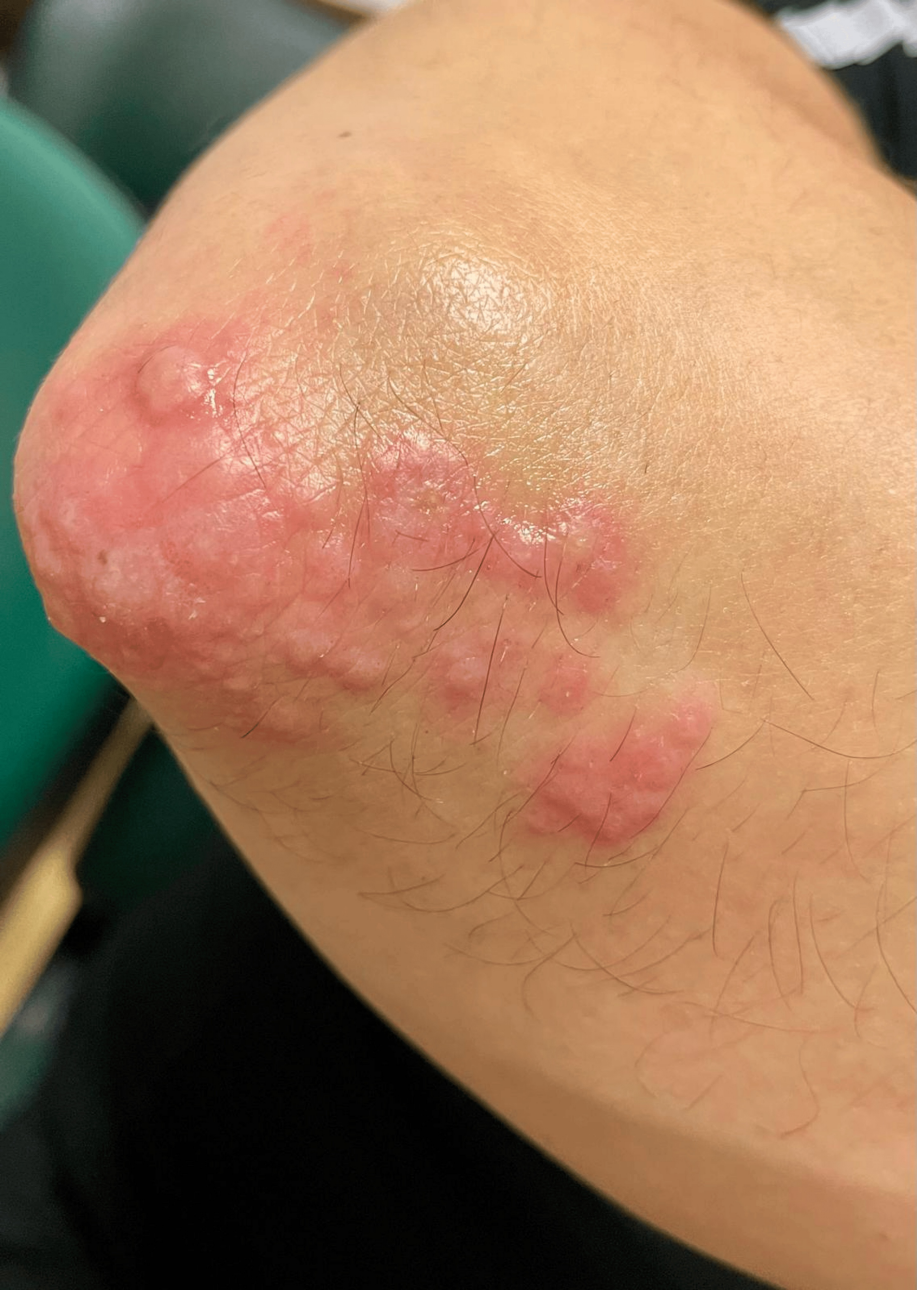
Vesicles, blisters and pustules on the extensor region of the right elbow.

A 48-year-old male, event organizer, single, without children, a smoking history of 33 pack-years, a history of plaque psoriasis for two years, regularly medicated and under control with betamethasone plus calcipotriol ointment, presented to the open clinic at a primary health care center with a one-day history of a skin rash on the hands, elbows, feet, and face, unresponsive to topical corticosteroid ointment. He also reported a prior two-week history of dry cough, odynophagia, rhinorrhea, and fever, which had spontaneously resolved. He denied risky contact with infected children or unprotected sexual relations. On physical examination, he had painful vesicles, blisters, and pustules on the perioral region, elbows, and palmoplantar regions of the hands and feet (Figures 1-3).

Given the clinical presentation, lesion location, and symptom progression, a clinical diagnosis of HFMD was made at the primary health care center, where laboratory tests were not available for diagnostic confirmation. The patient was discharged with reassurance of the clinical scenario, symptomatic measures, analgesia, and fusidic acid twice daily. After two days, he presented himself to the hospital emergency department due to worsening of the skin rash. He had new vesicles and pustules scattered on more locations, mainly on his face and ears, also painful, but with some lesions already resolving. A dermatological evaluation by a specialist was requested, who also made a clinical diagnosis of HFMD. For diagnostic confirmation and exclusion of other infections, blood tests were requested and revealed only a slight elevation of inflammatory parameters and a positive enterovirus A71 (Table 1).

**Table 1:** Blood test results. AST:aspartate transaminase; ALT: alanine transaminase; HBsAg: HBV surface antigen; VDRL: venereal disease research laboratory test; ASO:anti-streptolysin O.

Parameters	Value	Reference range
Hemoglobin	15 g/dL	13-16 g/dL
White blood cells	5900/µL	4000-11000/µL
Neutrophils	3650/µL	1500-8000/µL
Lymphocytes	1540/µL	800-4000/µL
C-reactive protein	3 mg/dL	0-0.2 mg/dL
Creatinin	1.0 mg/dL	0.7-1.2 mg/dL
AST	35 UI/L	0-40 UI/L
ALT	48 UI/L	0-50 UI/L
HIV 1, 2 antibodies	Negative	NA
HBsAg	Negative	NA
VDRL	Negative	NA
ASO	Negative	NA
Coxsackievirus A16	Negative	NA
Enterovirus A71	Positive	NA

He was discharged with a confirmed diagnosis of HFMD due to enterovirus A71 and advised to continue the previously prescribed treatment. Due to subsequent worsening of the plaque psoriasis, which was only responding partially to the topical therapy after the infection, the patient began follow-up in a dermatology consultation and started phototherapy with psoralen and ultraviolet A radiation. Subsequent information about the treatment efficacy is unknown because the patient moved to another location.

## Discussion

This case represents an adult patient who presented with skin lesions following a respiratory illness of suggestive viral etiology (cough, odynophagia, fever, rhinorrhea), which had resolved in the previous two weeks. Several factors challenged the diagnosis in this patient. The patient had a history of plaque psoriasis and did not exhibit any extracutaneous symptoms at the time of presentation. Some lesions also had atypical locations (e.g., elbows ), possibly due to the use of topical immunosuppressive therapy. Additionally, the epidemiological source of the infection was unclear. The patient was unaware of any contact with an infected person and denied frequent contact with the pediatric population. These factors contributed to an extensive differential diagnosis, which caused further concern for the patient and the need for diagnostic confirmation in a hospital setting.

The main differential diagnoses to consider are viral rashes, bacterial rashes, and skin conditions due to systemic pathology. Among infectious causes, monkeypox, chickenpox, and syphilis are the primary considerations. For monkeypox, there were no anogenital or perioral lesions, and the lesions did not evolve into pseudo-pustules. Regarding chickenpox, there were no lesions in various stages (blisters, ulcers, scabs) or trunk lesions. In secondary syphilis, most patients have enlarged lymph nodes in multiple locations (cervical, axillary, and inguinal). Also, there is usually a widespread rash affecting the trunk, which the patient did not show. 

HFMD usually presents with fever and vesicular lesions on the hands and feet and inside the mouth [3]. In adults, the symptoms can be atypical or severe, with higher fever and lesions on other parts of the body such as the arms, legs, and trunk [4]. Diagnosing atypical cases often depends on a thorough clinical history and physical examination, supported by diagnostic tests like serology or nucleic acid amplification [7]. Treatment focuses on relieving symptoms, with an emphasis on hydration and pain management [7]. The prognosis is generally good, with most cases resolving within one to two weeks and recurrences being rare. However, serious complications can occur, including painful stomatitis, pulmonary edema, myocarditis, interstitial pneumonia, and pancreatitis related to coxsackievirus [6,8]. As presented, there is also the possibility of worsening or exacerbating of chronic skin conditions, sometimes manifesting Koebner phenomenon, although there are few cases described in the literature in relation to HFMD in patients with psoriasis [7]. Patients with an underlying dermatological pathology may require more rigorous monitoring, sometimes at hospital level care, after the resolution of the infectious condition due to the need for therapeutic adjustment, whether for maintenance or crisis.

## Conclusions

This case report emphasizes the need for healthcare providers to consider HFMD in adults with unexplained exanthems and oral symptoms, especially when chronic dermatologic diseases are present due to the risk of exacerbation. Atypical presentations, though rare, require accurate diagnosis for appropriate management. Further research can enhance understanding and early treatment of HFMD in adults.

## References

[1] Flipo R, Isnard C, Coutard A, Martres P, Dumas M, Blum L, Begon E (2020). Atypical hand, foot and mouth disease in adults: a note on 6 cases]. Ann Dermatol Venereol.

[2] Di Prinzio A, Bastard DP, Torre AC, Mazzuoccolo LD (2022). Hand, foot, and mouth disease in adults caused by Coxsackievirus B1-B6. An Bras Dermatol.

[3] Guerra AM, Orille E, Waseem M (2023). Hand, Foot, and Mouth Disease. StatPearls [Internet].

[4] Ramirez-Fort MK, Downing C, Doan HQ, Benoist F, Oberste MS, Khan F, Tyring SK (2014). Coxsackievirus A6 associated hand, foot and mouth disease in adults: clinical presentation and review of the literature. J Clin Virol.

[5] Afonso C, Almeida A (2023). Hand, foot, and mouth disease in adults. Cureus.

[6] Gomes S, Santos S, Ferreira Maia I, Verissimo R, Carvalho T (2023). Hand-foot-mouth disease in an adult. Cureus.

[7] Cole DW, Wang B, Fullen DR, Helfrich YR (2022). Psoriasis coxsackium. JAAD Case Rep.

[8] Broccolo F, Drago F, Ciccarese G (2019). Severe atypical hand-foot-and-mouth disease in adults due to coxsackievirus A6: clinical presentation and phylogenesis of CV-A6 strains. J Clin Virol.

